# The causal relationship between gut microbiota and neuroblastoma: a bidirectional Mendelian randomization analysis and meta-analysis

**DOI:** 10.1128/spectrum.03656-23

**Published:** 2024-02-27

**Authors:** Zexin Zhang, Dongting Li, Fengxi Xie, Haibo Zhang

**Affiliations:** 1 The Second Clinical School of Guangzhou University of Chinese Medicine, Guangzhou, China; 2 The Affiliated Guangzhou Hospital of TCM of Guangzhou University of Chinese Medicine, Guangzhou, China; 3 Maoming Hospital of Guangzhou University of Chinese Medicine, Maoming, China; 4 The Second Affiliated Hospital of Guangzhou University of Chinese Medicine, Guangzhou, China; 5 Guangdong Key Laboratory of Clinical Research of Chinese Medicine, Guangzhou, China; 6 Guangdong Joint Laboratory of Guangdong, Hong Kong and Macao Chinese Medicine and Immune Diseases, Guangzhou, China; 7 State Key Laboratory of Wet Certificate of Chinese Medicine Jointly Built by the Province and the Ministry, Guangzhou, China; Tianjin University, Tianjin, China

**Keywords:** causal relationship, gut microbiota, neuroblastoma, Mendelian randomization analysis

## Abstract

Neuroblastoma (NB) is a type of neuroectodermal tumor that originates from primitive sympathetic ganglion cells. Although many risk factors contributing to the occurrence of NB have been reported in recent years, the role of the gut microbiota in its development remains unclear. A bidirectional Mendelian randomization (MR) analysis was conducted to elucidate the causal relationship between the gut microbiota and NB. In the MR analysis, we employed the inverse-variance weighted (IVW) method as the primary criterion for assessing causality, while also utilizing three additional approaches, including MR-Egger, weighted median model, and weighted mode, for comprehensive evaluation. For gut microbiota that were causally associated with NB, a reverse MR was also used to assess the stability of this causal relationship. Finally, we also used external cohorts for validation and performed a meta-analysis of the results. The IVW results indicated a causal relationship between six gut microbiota and NB. Among the six gut microbiota, genus *Lachnospiraceae* [IVW odds ratio (OR): 2.66, 95% confidence interval (CI): 1.09–6.51, *P* value: 0.03] exhibited a detrimental effect against NB. On the other hand, the class *Actinobacteria* (IVW OR: 0.24, 95% CI: 0.07–0.77, *P* value: 0.02), the family *Bifidobacteriaceae* (IVW OR: 0.40, 95% CI: 0.17–0.96, *P* value: 0.04), the genus *Desulfovibrio* (IVW OR: 0.50, 95% CI: 0.25–0.97, *P* value: 0.04), the genus *Bifidobacterium* (IVW OR: 0.39, 95% CI: 0.16–0.92, *P* value: 0.03), and the genus *Howardella* (IVW OR: 0.55, 95% CI: 0.31–0.97, *P* value: 0.04) displayed a protective effect on NB. A reverse MR analysis did not reveal a causality between NB and the six gut microbiota. Meta-analysis showed that genus *Bifidobacterium* (meta OR: 0.41, 95% CI: 0.22–0.75, *P* < 0.01) and genus *Lachnospiraceae* (meta OR: 2.20, 95% CI: 1.01–4.79, *P* < 0.05) were still significant.

**IMPORTANCE:**

Bidirectional Mendelian randomization was used to explore the causality between gut microbiota and neuroblastoma (NB). The results showed that there is a causal relationship between the six gut microbiota and NB, of which two gut microbiota were further confirmed in the meta-analysis. This may provide a new perspective on the prevention and treatment of NB.

## INTRODUCTION

Neuroblastoma (NB) is a malignant tumor originating from primitive neural crest cells outside the skull, and it predominantly occurs during childhood (1). NB originates from the sympathetic nervous system in the abdomen or the chest. Its most common occurrence is in the adrenal glands (2). NB accounts for 8%–10% of all childhood cancers, but it results in a mortality rate of 12%–15% among all pediatric malignancies (3). Epidemiological data also indicated an increasing trend in the incidence of NB (4). NB is a complex disease characterized by a wide range of clinical symptoms (5). The clinical presentation of NB includes abdominal distension, abdominal pain, persistent diarrhea, and irritability. Classic symptoms such as fever, pain, and irritability are associated with metastatic NB (6). Different ages of onset, tumor locations, and varying degrees of tissue differentiation can result in significant prognostic variability (2, 5). Some NB cases can spontaneously regress or transform into benign tumors, while others may progress relentlessly and even lead to patient mortality. High-risk NB patients, constituting approximately 50% of all NB cases, often present with unresectable primary lesions or widespread metastases (7). Even with comprehensive treatments including radiation therapy and chemotherapy, the 5-year survival rate for high-risk pediatric NB patients remains below 50% (8). Furthermore, the 5-year survival rate for refractory or recurrent NB is only 20% (9), significantly impacting the lives of affected children. This is why NB is referred to as the “king of childhood tumors,” placing a heavy financial burden on families. The current treatment phases for high-risk NB are induction, consolidation, and maintenance. Induction chemotherapy is used to reduce tumors by making them smaller and to decrease the risk of tumor metastasis through chemotherapy and surgery (10). However, tumor heterogeneity, drug resistance, and severe toxicity make the effect of NB treatment unsatisfactory (1). Therefore, new treatment strategies are urgently needed for NB patients.

The pathogenesis of NB has not yet been elucidated. However, relevant studies have shown that the onset of NB does not seem to have a direct cause. It requires multiplicity and the synergy of various effects to initiate tumorigenesis (11). The intestinal flora is an ecosystem composed of bacteria, viruses, archaea, fungi, and protozoa living in the gastrointestinal tract. It is also often called the second genome. More and more studies have shown that there is a close connection between microorganisms and different diseases, such as tumors (12) and type 2 diabetes (13). Therefore, the microbiota is a potentially promising way to prevent and treat diseases (14). NB can synthesize and secrete large amounts of catecholamines, and the determination of catecholamines and their metabolites is helpful in the diagnosis of NB. Systemic symptoms caused by catecholamines, such as fever and irritability, can be observed in NB patients (15). The intestinal flora can also produce a series of neuroactive substances such as catecholamines and acetylcholine. Excessive secretion of vasoactive intestinal peptide (VIP) by NB can lead to the occurrence of intractable diarrhea (16), and reports suggest that inhibiting VIP-producing enteric neurons can lead to a decrease in the abundance of bacteria associated with *Bifidobacterium* in the intestinal tract (17). Castellani et al. (18) found that in a murine model of NB similar to adult cancer, there was a reduction in the abundance of *Firmicutes* in the ileum of NB mice. In contrast, the abundance of *Enterobacteriaceae*, *Clostridium UBA1819*, and *Lachnospiraceae UCG013* increased. The tumor microenvironment in NB can inhibit tumor-infiltrating lymphocytes, natural killer cells, and dendritic cells, and it can also elevate the levels of regulatory T cells. Meanwhile, the gut microbiota can modulate adaptive immunity by maintaining a balance between effector T cells and regulatory T cells (19). This suggests a potential correlation between NB and the gut microbiota. However, due to the lack of evidence from randomized controlled trials, it is still unclear whether there is a definitive causal relationship between the gut microbiota and NB.

Mendelian randomization (MR) is a causal inference method based on genetic variation. MR utilizes the random segregation of alleles during the formation of gametes in meiosis, and because these genetic variations precede the progression of diseases and are not influenced by postnatal lifestyle and environmental factors, MR can minimize the impact of confounding factors (20). In this study, a large-scale Genome-Wide Association Study (GWAS) data set was used to investigate potential causal relationships between the gut microbiota and NB through a two-sample MR analysis. This research aims to provide an additional clinical approach for NB treatment: targeting specific gut microbiota to achieve therapeutic goals in NB.

## MATERIALS AND METHODS

### Data sources

All the genetic data on gut microbiota and NB were sourced from the GWAS summary data available on the IEU Open GWAS Project website (https://gwas.mrcieu.ac.uk/). The IEU Open GWAS Project serves as an openly accessible database containing extensive genetic association data for querying or downloading. We performed searches using the keywords “Gut microbiota abundance” and “neuroblastoma” within the “Trait contains” search field. Gut microbiota entries categorized as “unknown” were excluded from our analysis.

We obtained data for 211 gut microbiota, excluding 15 that were labeled as unknown. In total, 196 gut microbiota were included in the analysis of this study (21). These gut microbiota were classified into phylum, order, genus, family, and class. Almost all samples were derived from the European population. This study included a total of 18,340 individuals in 24 cohorts, one of which included 13,266 individuals in the European cohort. The NB data were sourced from Capasso et al. (22) published in 2013, comprising 4,881 samples and 468,788 single nucleotide polymorphisms (SNPs), including 1,627 patient samples and 3,254 control samples.

### Selection of instrumental variables

SNPs that satisfy the three core assumptions of MR were used as instrumental variables (IVs) for exposure and outcome. The three core assumptions of MR were as follows: the relevance assumption, the independence assumption, and the exclusion restriction assumption. The relevance assumption typically requires that the chosen IVs be highly correlated with the exposure. In this study, to screen for additional IVs, we set the *P* value for gut microbiota at 1E−5 and applied conditions for linkage disequilibrium, which refers to the situation where the association between two or more genes exceeds random expectations, indicating that the alleles of these genes are not independently inherited. In the context of MR analysis, the presence of linkage disequilibrium can lead to unreliable results. Therefore, we set the criteria for filtering at kb = 10,000 and *r*^2^ = 0.001. Furthermore, to enhance the reliability of these IVs, we employed *F*-tests to validate each SNP, aiming to exclude any potential weak IVs. For IVs with an *F*-test greater than 10, they were considered to meet this criterion. The formula for the *F*-test is as follows:


$$F={\left(\frac{Beta}{SE}\right)}^{2}$$

In this formula, the beta value represents the effect size of the exposure instrument, SE represents the standard error of the exposure instrument, and the *F* value is obtained by squaring the result of beta divided by SE (23).

Furthermore, MR analysis typically requires satisfying the independence assumption and exclusion restriction assumption. The independence assumption requires that the chosen IVs be unrelated to any confounding factors that affect the association between exposure and outcome. The exclusion restriction assumption demands that IVs do not influence the outcome except through their impact on exposure. To fulfill these assumptions, we implemented pleiotropy tests and heterogeneity tests. Pleiotropy is not allowed in MR analysis, and we utilized the methods of MR Presso and MR-Egger to conduct pleiotropy tests. For heterogeneity, besides excluding SNPs with heterogeneity, we employed a random effects model to provide a more accurate assessment of the results. We used the heterogeneity function of the TwoSampleMR package in R language 4.3.1 for heterogeneity tests. Additionally, we conducted a leave-one-out sensitivity analysis to evaluate the impact of each SNP on the outcome. Finally, for those gut microbiota causally associated with NB, we performed a reverse MR analysis.

### Mendelian randomization analysis

Inverse-variance weighted (IVW) was used as the primary method to assess the results of MR. In addition, we also employed MR-Egger, weighted median model, and weighted mode simultaneously as supplemental methods. The advantage of IVW lies in its ability to provide more robust results, even if there is one ineffective SNP in the instrumental variable set, the results may still be biased. MR-Egger and IVW are similar algorithms; however, MR-Egger is an improvement over IVW. This is because MR-Egger incorporates an intercept term in the regression model to detect and correct for pleiotropy effects. The weighted median model and weighted mode share similarities, with the key distinction being that the weighted median model employs the concept of the median. It sorts the effect values of different SNPs and selects the median as the estimated causal effect. In contrast, the weighted mode model utilizes the reciprocal of the result variance as weights, meaning that SNPs with smaller variances contribute more significantly to the estimation.

### Validation based on external cohorts and meta-analysis

To further verify the reliability of MR analysis results, we used external cohorts to verify the positive results of MR. For the intestinal flora with positive results, we obtained data sets from different sources in the IEU Open GWAS Project for verification. It is worth noting that in order to make the results more robust, we eliminated these data sets that are not at the same level in bacterial classification. For the validation process on external cohorts, we performed the analysis using the same parameters and procedures used for MR analysis as described above. For the analyzed results, we performed a meta-analysis to merge effect sizes. *I*^2^ was used to assess the heterogeneity of the meta-analysis. For *I*^2^ ≤ 50% and *P* value ≥0.05, we adopt a fixed effects model. For *I*^2^ > 50% and *P* value <0.05, we used the random effects model.

## RESULTS

### Characteristics of SNPs

Based on the aforementioned selection criteria, we extracted SNPs from the exposure for the current analysis. A total of 224, 434, 512, 486, 498, 280, and 125 SNPs were, respectively, obtained from the class, family, genus1, genus2, genus3, order, and phylum for the gut microbiota. Additionally, from the outcome “Neuroblastoma,” 19, 31, 62, 59, 51, 28, and 21 SNPs were extracted for analysis (Table S1). In this study, no SNPs with an *F* value <10 were found, indicating that the IVs we used were effective tools.

### Mendelian randomization analysis

**Fig 1:**
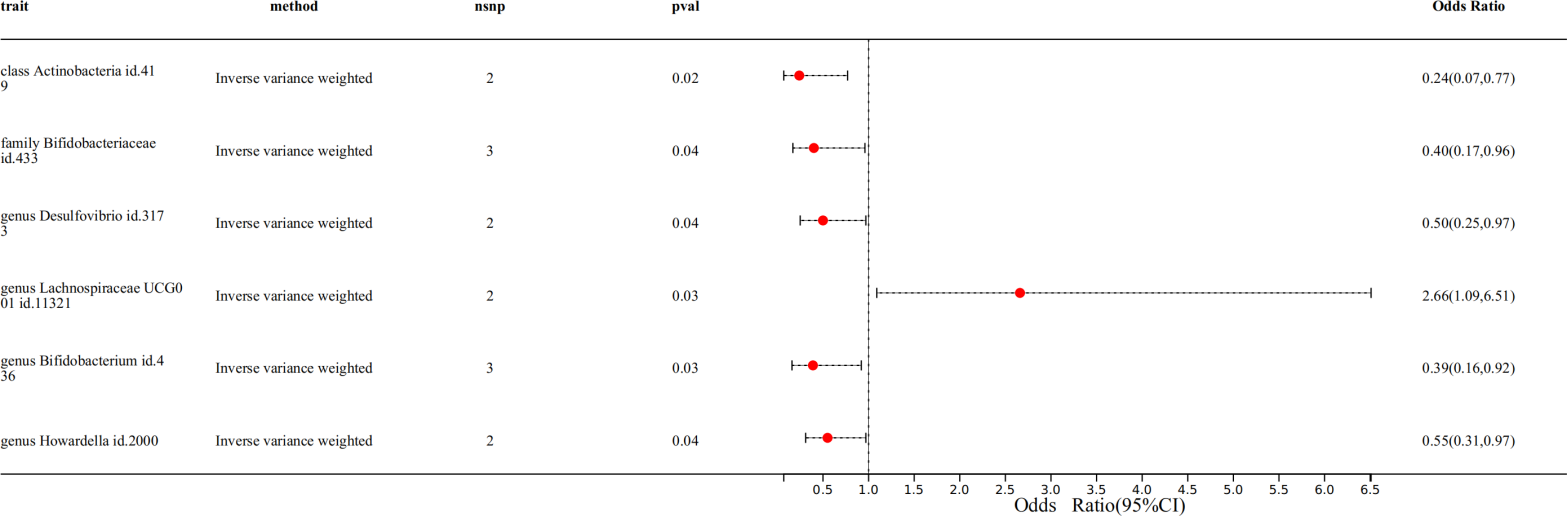
The IVW results of six gut microbiota against NB.

The IVW results indicated a causal relationship between six gut microbiota and NB. Among the six gut microbiota, genus *Lachnospiraceae* [IVW odds ratio (OR): 2.66, 95% confidence interval (CI): 1.09–6.51, *P* value: 0.03] exhibited a detrimental effect against NB. On the other hand, the class *Actinobacteria* (IVW OR: 0.24, 95% CI: 0.07–0.77, *P* value: 0.02), the family *Bifidobacteriaceae* (IVW OR: 0.40, 95% CI: 0.17–0.96, *P* value: 0.04), the genus *Desulfovibrio* (IVW OR: 0.50, 95% CI: 0.25–0.97, *P* value: 0.04), the genus *Bifidobacterium* (IVW OR: 0.39, 95% CI: 0.16–0.92, *P* value: 0.03), and the genus *Howardella* (IVW OR: 0.55, 95% CI: 0.31–0.97, *P* value: 0.04) showed a protective effect on NB (Fig. 1; Table S2). The forest plot shows the distribution of the effect size of each SNP, using the method of IVW and MR-Egger (Fig. S1). The scatter plot illustrates the overall trend of the impact of exposure factors on outcome factors. When the overall trend is from the bottom left to the top right, it indicates a hazardous trend of exposure factors on outcome factors. Conversely, if the overall trend is from the top left to the bottom right, it suggests a protective trend of exposure factors on outcome factors (Fig. S2). A reverse MR analysis did not reveal a causal relationship between NB and the aforementioned six gut microbiota.

Although the funnel plot did not clearly demonstrate whether SNPs in each individual analysis were symmetrically distributed, this may be due to the limited number of SNPs retained for analysis (Fig. S3). However, the heterogeneity test demonstrated that these SNPs did not exhibit heterogeneity (Table S3). The Egger intercept was very close to 0, and its *P* value was consistently >0.05, indicating the absence of SNPs with pleiotropic effects in this study (Table S4). Sensitivity analysis using the leave-one-out method revealed that no SNP significantly influenced the results, further confirming the robustness of the findings (Fig. S4).

### Validation based on external cohorts and meta-analysis

**Fig 2:**
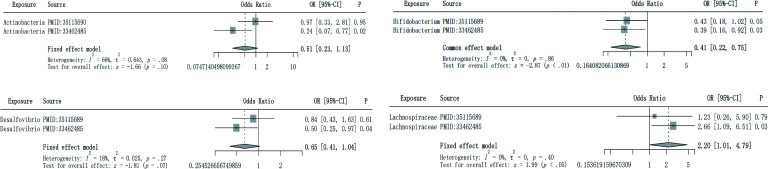
Meta-analysis of four gut microbiota against NB.

Data sets of five intestinal flora used for the validation of MR analysis results were obtained from the IEU Open GWAS Project, consistent with this study at the taxonomic level of intestinal flora to ensure good homogeneity. Since the family *Bifidobacteriaceae* failed to obtain the same SNP in the outcome, four data sets of intestinal flora were finally left for the meta-analysis. These data sets came from two different large-scale studies, which contained 5,959 and 7,738 samples, respectively. The populations were all from Europe, and their internal homogeneity was good (24, 25). The former study population came from the FR02 study, which included men and women aged 25–74 years from six geographical regions in Finland. The latter study was from the LifeLines multidisciplinary prospective population-based cohort study, in which all participants were invited to voluntarily participate in a parallel project, the DMP, during a follow-up periodty. tyThe goal of this project was to evaluate the impact of different exposures and lifestyles on the composition of the gut microbiota. 58.1% of them were female, aged 8–84 years old (average 48.5 years old). The heterogeneity test showed that there was no data set with *I*^2^ > 50% and *P* value <0.05, so a fixed effects model was used for meta-analysis. Meta-analysis results showed that the genus *Bifidobacterium* (meta OR: 0.41, 95% CI: 0.22–0.75, *P* < 0.01) has a protective effect on NB, and the genus *Lachnospiraceae* (meta OR: 2.20, 95% CI: 1.01–4.79, *P* < 0.05) has a pathogenic effect on NB. The genus *Actinobacteria* (meta OR: 0.51, 95% CI: 0.23–1.13, *P* = 0.10) and the genus *Desulfovibrio* (meta OR: 0.65, 95% CI: 0.41–1.04, *P* = 0.07) have potential protective effects on NB (Fig. 2).

## DISCUSSION

This study represents the first-ever bidirectional MR analysis of the gut microbiota and NB. The research findings demonstrated that there was a causal relationship between a total of six specific gut microbiota and NB. Some previous observational studies have explored the relationship between gut microbiota and NB. The changes in the gut microbiota of NB mouse models were first reported by Castellani et al., showing a decreasing trend in the abundance of *Firmicutes* and an increasing trend in the abundance of *Bacteroidetes*, *Deferribacteres*, and *Tenericutes* in the microbiota (18). Meanwhile, in another mouse model, it was concluded that there was a significant decrease in the abundance of *Lactobacillus* (26). The above studies suggested a potential correlation between the gut microbiota and NB, but the results were not consistent, and further evidence is needed to clarify their relationship. As of now, there have been no MR analysis studies exploring the causal relationship between gut microbiota and NB. The results of this study could provide a novel treatment strategy for NB.

Our research has revealed that there is a causal relationship between six specific gut microbiota and NB. Among them, the family *Lachnospiraceae* is the sole risk factor for NB. According to reports, the abundance of *Lachnospiraceae* is positively correlated with the expression of brain-derived neurotrophic factor (BDNF) (27). As a member of the neurotrophic factor family, BDNF plays a crucial role in maintaining the development of the peripheral sympathetic and neural crest-derived sensory neurons (28). However, on the other hand, BDNF’s binding to the tyrosine kinase receptor TrkB is highly expressed in many malignant tumors and is associated with poor prognosis. The mouse experiments conducted by Hua et al. demonstrated that BDNF/TrkB promotes the metastasis and invasion of NB cells through the PI3K/Akt/mTOR and MAPK pathways (28).

In addition to the family *Lachnospiraceae*, we have also identified five other gut microbiota as protective factors against NB. *Actinobacteria* and their bioactive molecules have been a recent focus of research. Studies have shown that 90 strains of *Actinobacteria* can be isolated from large algae, among which the extract from 24 strains of *Actinobacteria* effectively reduces the viability of NB cells (29). It exhibits a certain inhibitory effect on the growth of NB cells.

Order *Bifidobacteriales* and its subgroups, family *Bifidobacteriaceae*, and genus *Bifidobacterium* are all protective factors against NB. In the progression of most NBs, the PI3K/Akt/mTOR pathway is an important activated pro-survival signaling pathway (30). The activation of this pathway can promote mutations or overexpression of growth factor receptors or their ligands, leading to aberrant receptor tyrosine kinase activity in NB (31). On the other hand, during the development of NB cells, overexpression of MYCN is required, and the PI3K/Akt/mTOR pathway effectively maintains the stability of MYCN (1). *Bifidobacterium*, a well-known probiotic in the human intestinal tract, can produce galactose through lactose fermentation. Galactose is a component of brain glycosides in the nervous system and is closely associated with the rapid growth of the brain after birth. Relevant studies suggest that galactose can effectively inhibit the proliferation and growth of cancer cells activated by the AKT signaling pathway (32). Moreover, mouse experiments conducted by Li Wang and colleagues have effectively demonstrated that a combination of *Lactobacillus acidophilus* and plant-derived *Lactobacillus* can inhibit the PI3K/AKT pathway (33). In addition, Henrick et al. found that infants with a high abundance of *Bifidobacterium* had elevated levels of Treg-related cytokine interleukin-27 (IL-27). IL-27 is an immune-modulating cytokine expressed by activated macrophages and dendritic cells. It inhibits immune responses within the tumor microenvironment through multiple mechanisms, playing a role in preventing cancer development (34). Therefore, the protective effects of order *Bifidobacteriales*, family *Bifidobacteriaceae*, and genus *Bifidobacterium* on NB may primarily manifest in two aspects: first, *Bifidobacterium* and its metabolic byproducts can inhibit the progression of NB by suppressing the PI3K/Akt/mTOR signaling pathway; second, *Bifidobacterium* influences the immune response within the tumor microenvironment by promoting the expression of IL-27, thereby preventing the development of NB.

The genus *Desulfovibrio* showed protective factors against NB. According to research reports, *Desulfovibrio* is closely associated with the production of hydrogen sulfide (H_2_S) (35). As *N*-acetyl-L-cysteine (NAC) serves as a precursor for the synthesis of H_2_S, researchers have found that treating NB cells with NAC inhibits the proliferation of NB cells (36). Therefore, the genus *Desulfovibrio* may achieve the inhibition of NB progression by promoting the production of NAC.

The genus *Howardella* being identified as a protective factor for NB is interesting, especially considering that the relationship between *Howardella* and NB has not been explored. This may suggest a new direction for future research on NB.

### Conclusions

In summary, this study identified a causal association between six gut microbiota and NB through a bidirectional MR analysis. This finding may potentially offer a novel strategy for the prevention and treatment of NB. Of course, this study also has some limitations, such as the small number of IVs ultimately included and some analyses that did not undergo sensitivity analysis. The relationship between gut microbiota and NB is highly complex, and further validation is still required through additional randomized controlled trials and experiments.

## Data Availability

The IEU Open GWAS Project database supplied all the necessary data for this study.

## SUPPLEMENTAL MATERIAL

Fig. S1 spectrum.03656-23-s0001.tif Distribution of the effect size of each SNP based on the Egger and IVW.

Fig. S2 spectrum.03656-23-s0002.tif Overall trend of the impact of exposure factors on outcome factors.

Fig. S3 spectrum.03656-23-s0003.tif Heterogeneity test based on funnel plot.

Fig. S4 spectrum.03656-23-s0004.tif Sensitivity analysis using leave-one-out method.

Table S1 spectrum.03656-23-s0005.xls IVs used for analysis in this study.

Table S2 spectrum.03656-23-s0006.xls Results of MR analysis between 6 gut microbiota and NB.

Table S3 spectrum.03656-23-s0007.xls Heterogeneity test between 6 gut microbiota and NB.

Table S4 spectrum.03656-23-s0008.xls Egger intercept between 2 gut microbiota and NB.

ASM does not own the copyrights to Supplemental Material that may be linked to, or accessed through, an article. The authors have granted ASM a non-exclusive, world-wide license to publish the Supplemental Material files. Please contact the corresponding author directly for reuse.
