# Supplementary figures and images for: The causal relationship between gut microbiota and neuroblastoma: a bidirectional Mendelian randomization analysis and meta-analysis
Source: Microbiol Spectr. 2024 Feb 27;12(4):e03656-23. doi: 10.1128/spectrum.03656-23 (PMC10986465; doi:10.1128/spectrum.03656-23)

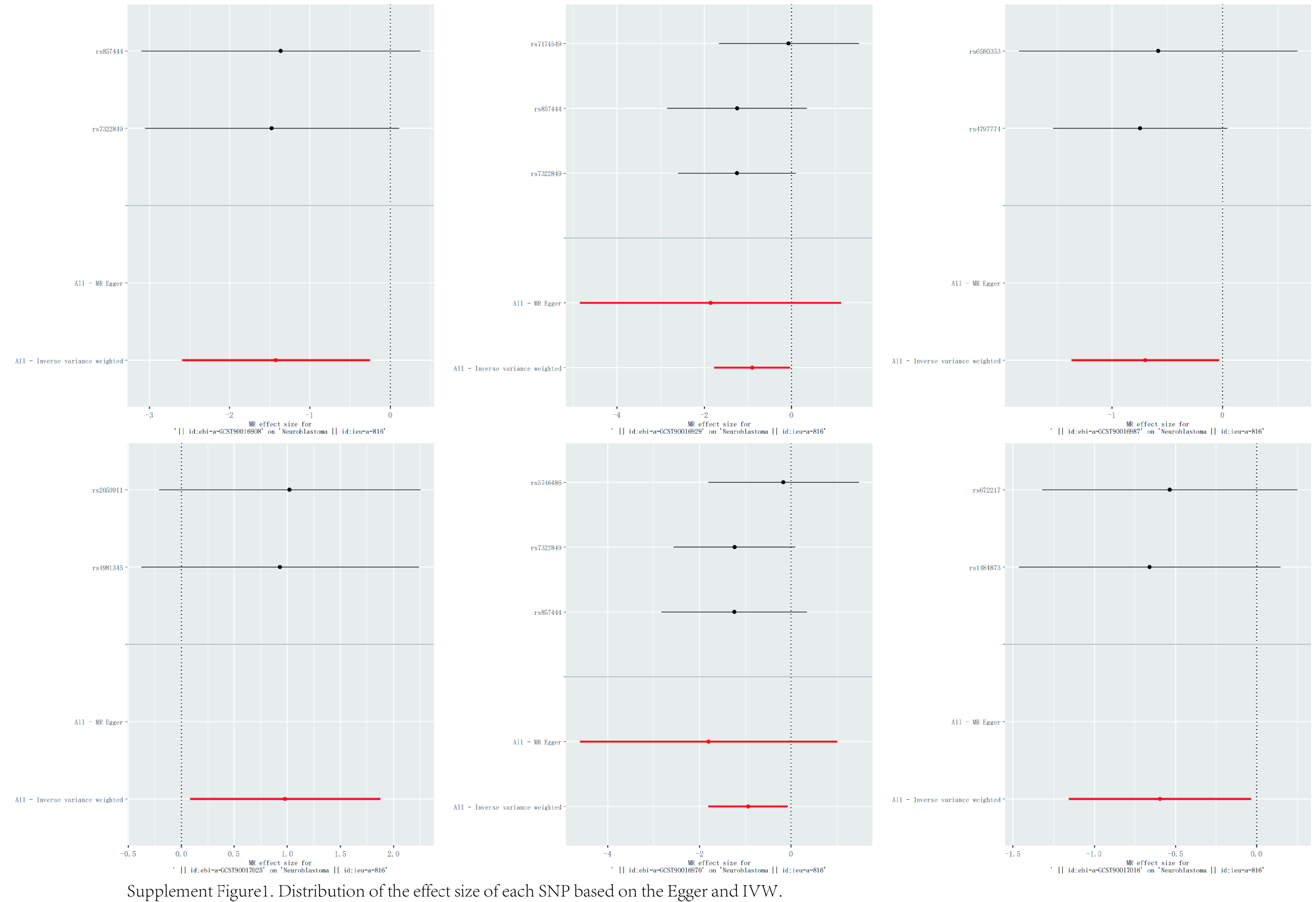

Supplement: Fig. S1 — Distribution of the effect size of each SNP based on the Egger and IVW. [file spectrum.03656-23-s0001.tif]

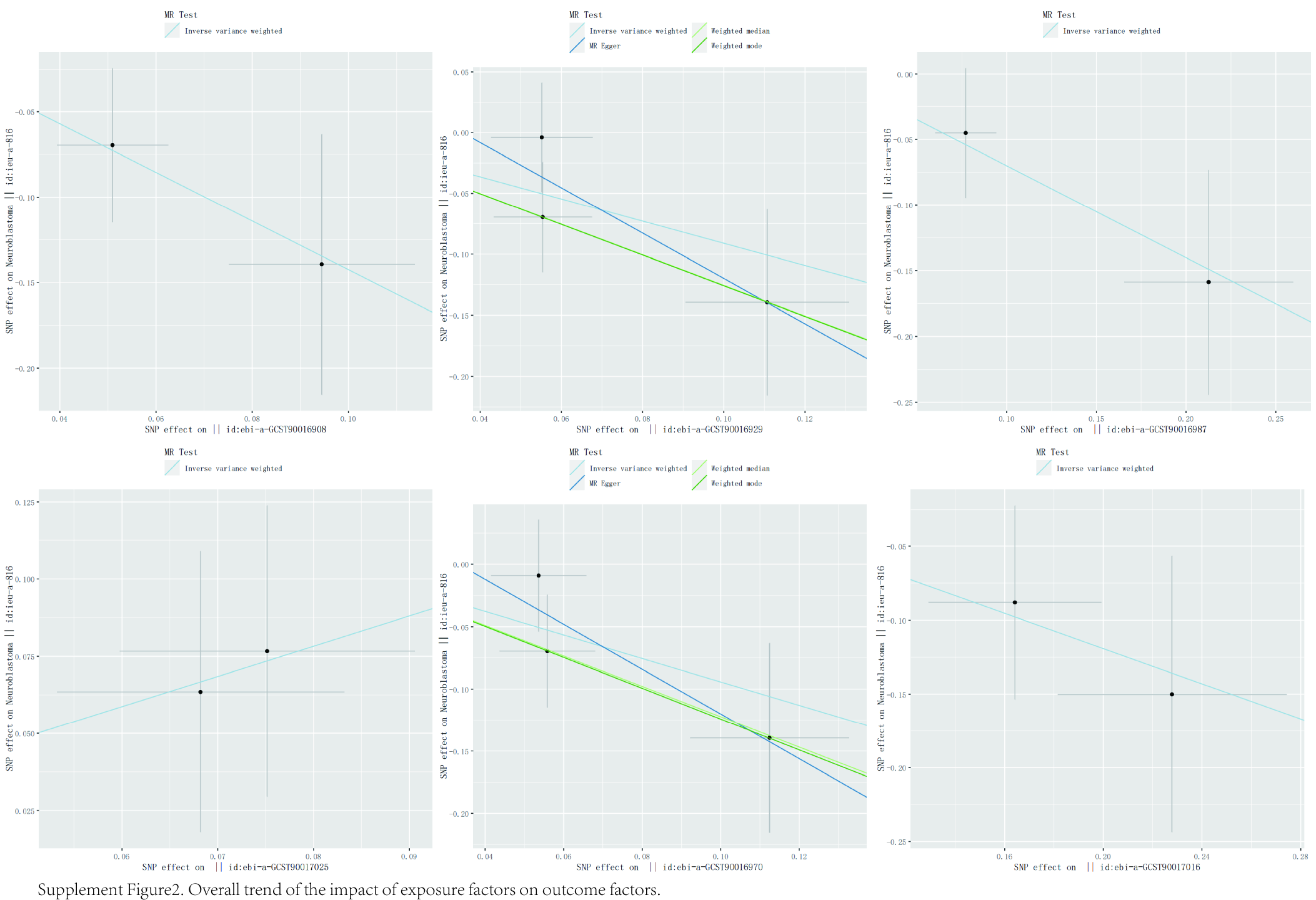

Supplement: Fig. S2 — Overall trend of the impact of exposure factors on outcome factors. [file spectrum.03656-23-s0002.tif]

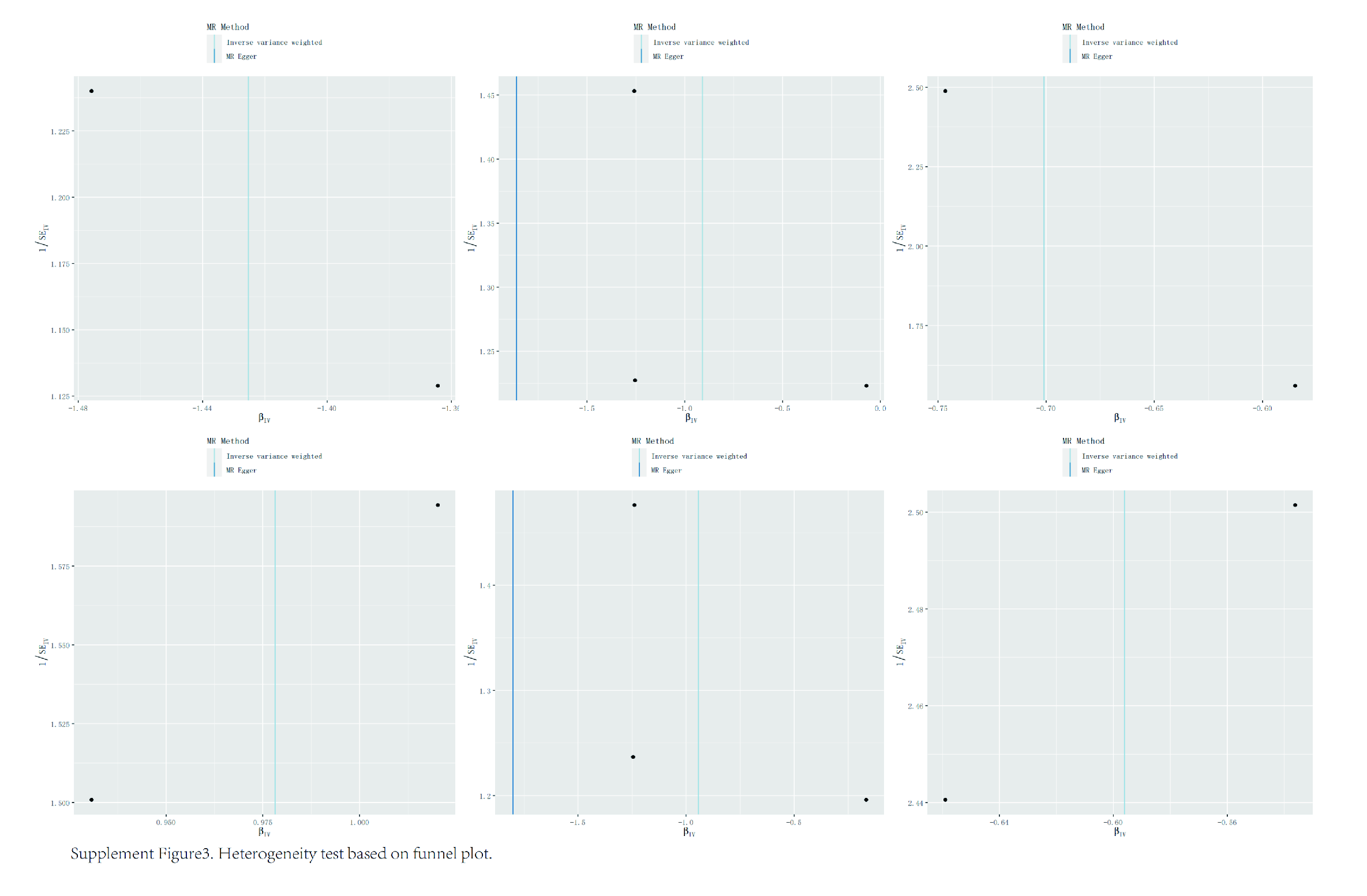

Supplement: Fig. S3 — Heterogeneity test based on funnel plot. [file spectrum.03656-23-s0003.tif]

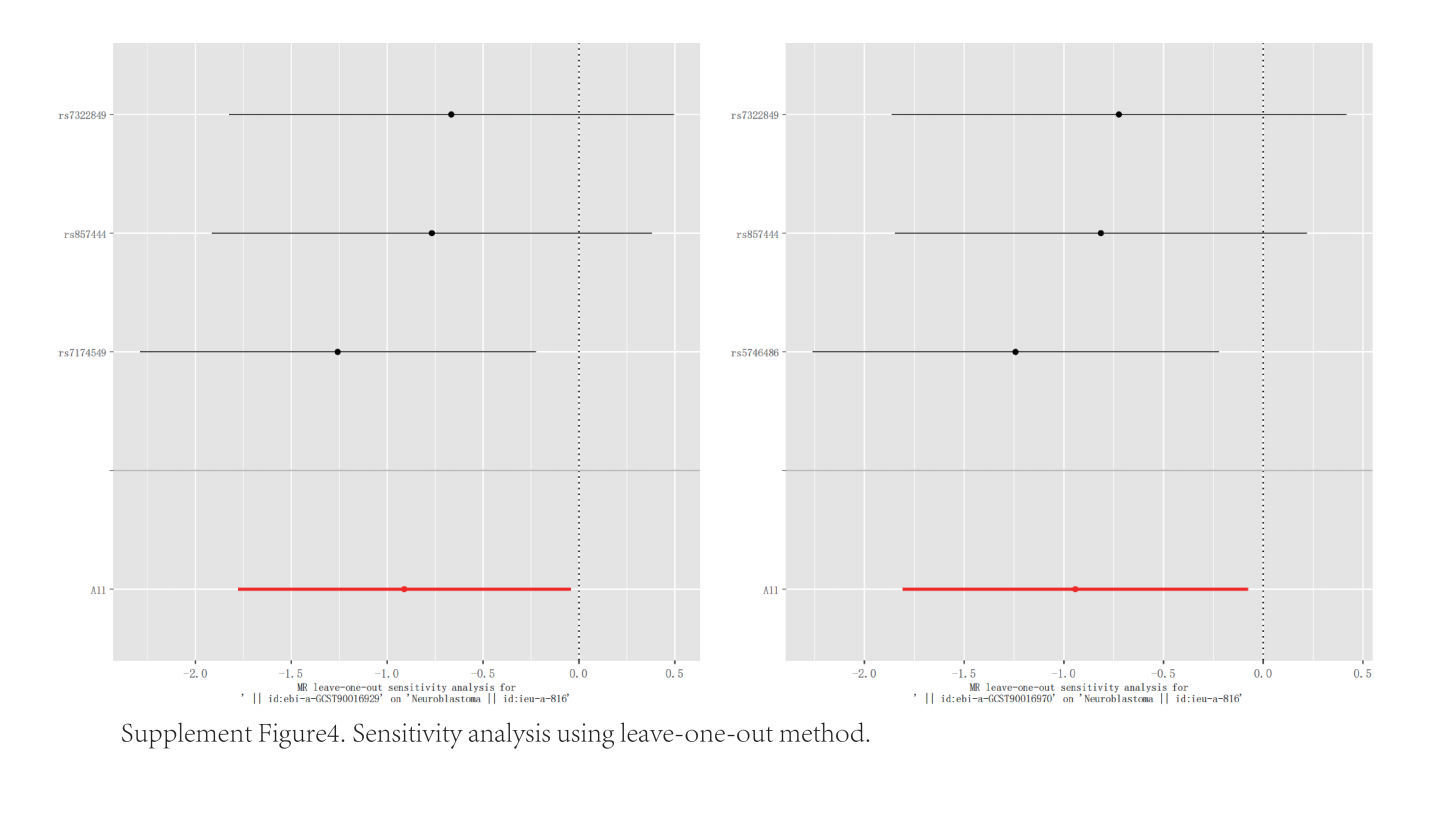

Supplement: Fig. S4 — Sensitivity analysis using leave-one-out method. [file spectrum.03656-23-s0004.tif]
